# Impact of metformin on disease control and survival in patients with head and neck cancer: a retrospective cohort study

**DOI:** 10.1186/s40463-019-0348-5

**Published:** 2019-07-25

**Authors:** Daniel J. Lee, Caitlin P. McMullen, Andrew Foreman, Shao Hui Huang, Lin Lu, Wei Xu, John R. de Almeida, Geoffrey Liu, Scott V. Bratman, David P. Goldstein

**Affiliations:** 1 Department of Otolaryngology Head and Neck Surgery/Surgical Oncology, University Health Network, Princess Margaret Cancer Center, University of Toronto, 610 University Ave 3-952, Toronto, ON M4V 2N8 Canada; 2 Department of Radiation Oncology, University Health Network, Princess Margaret Cancer Centre, University of Toronto, Toronto, ON Canada; 3 Biostatistics Department, Princess Margaret Cancer Centre and Dalla Lana School of Public Health, University of Toronto, Toronto, ON Canada

**Keywords:** Head and neck cancer, Diabetes, Metformin

## Abstract

**Objective:**

A number of in vitro and clinical studies have suggested potential antineoplastic effects of metformin. The impact of this medication on outcomes in head and neck cancer is less clear. Our aim was to determine the effect of metformin on outcomes within our large cohort of head and neck cancer patients with Type II Diabetes (T2DM).

**Study design:**

Retrospective cohort study.

**Setting:**

Tertiary Cancer Centre.

**Subjects and methods:**

A retrospective review of individuals with head and neck squamous carcinoma (HNSCC) and T2DM between January 2005 and December 2011 at Princess Margaret Cancer Centre was conducted. Medication history was obtained from surveys at initial presentation and electronic medical record review. Using Cox regression analyses, the association between metformin use and local, regional and distant failures was explored. Subgroup analyses were conducted for oral cavity, oropharynx and larynx.

**Results:**

A total of 329 HNSCC patients with T2DM were identified, including 195 metformin users and 134 non-metformin users. Patients were well-matched in terms of clinical, pathologic, and treatment factors. No difference in local, regional, or distant failure was observed between diabetic metformin users and diabetic non-metformin users for the entire cohort or within subgroup analysis for subsite. No difference between the two groups was observed for overall survival, recurrence-free survival, and disease-specific survival at 5 years.

**Conclusion:**

No association between metformin use and oncologic outcomes were observed in this large cohort of HNSCC patients. Multicenter, prospective studies may be needed to verify previous studies identifying a potential anti-neoplastic effect of this medication.

## Introduction

Metformin, an oral hypoglycemic agent, has been recognized for its potential antineoplastic effects. This low-cost, widely available, biguanide medication is indicated most commonly for the treatment of Type II diabetes mellitus (T2DM) and has a favorable adverse effect profile [1–3]. In vitro studies have demonstrated that metformin can potentiate the effects of chemotherapeutics and radiation as well as suppress tumor growth [4–8]. Clinically, metformin use in T2DM patients has been reported to be associated with improved oncologic outcomes in a number of cancers including breast, colorectal, prostate, thyroid, liver, and lung, compared to diabetic non-metformin users [9–14]. However, the results have been conflicting, with a recent meta-analysis of 11 randomized clinical trials involving 398 cancer types reporting no significant effect of metformin on all-cause mortality [14].

There have been a few studies assessing the impact of metformin use on outcomes in patients with head and neck squamous cell carcinoma (HNSCC) with mixed results [15, 16]. We have previously reported that the presence of T2DM alone does not adversely effect cancer survival outcomes among HNSCC patients managed at the Princess Margaret Cancer Center despite the presumed burden of diabetic comorbidity [17]. The objective of this study was to determine if metformin use in T2DM patients was independently associated with improved oncologic outcomes across the different subsites in HNSCC.

## Methods

### Study cohort and data acquisition

Following approval by the institutional Research Ethics Board, a retrospective chart review was performed of patients with a newly diagnosed, previously untreated, squamous cell carcinoma of the oral cavity, oropharynx, larynx and hypopharynx managed in our institution between January 2005 and December 2011. Patients were eligible for inclusion if they were > 18 years of age, had a diagnosis of T2DM and received curative intent treatment. Patients were identified through the institutional Cancer Registry and from a radiation oncology prospective anthology of outcomes database (Anthology Database) of all radiation oncology patients in our institution [18].

Sociodemographic information, treatment details, pathologic data and outcomes were obtained from the Anthology Database and supplemented by chart review. The T2DM patients identified in our previous study on outcomes of patients with HNSCC with and without T2DM were included in the current study [17]. Co-morbidities and medication history (primarily metformin use and combination oral medications that contained metformin) were obtained from chart review which were then cross-referenced with two additional databases, if available: (1) The Clinical Ambulatory Information System (CAIS) electronic preoperative anesthesia record for patients managed with primary surgery and (2) the head and neck translational research database, which has aimed to prospectively enroll all patients with a new diagnosis of HNSCC at Princess Margaret Cancer Center. The latter database requires patients complete a detailed medical and medication history, including self-reported diabetes. All patients were staged using the 7th edition of the *AJCC/UICC Cancer Staging Manual* [19]. Treatment details at our center have been described in the previous publication [17].

### Statistical analysis

Patients using metformin at the time of their HNSCC diagnosis were classified as Type 2 Diabetic Metformin positive (T2DMM+). Summary statistics were provided for clinical and demographic variables. Baseline patient characteristics were compared between metformin and non-metformin users. In an attempt to better delineate the effects of metformin, we focused on the T2DM group only, which is also in keeping with other head and neck studies [15, 16]. Differences between the two groups were examined by chi-square (χ^2^) test and T test for categorical variables and continuous variables, respectively. The clinical end points were control rates and survival. Overall survival (OS) was defined as time from date of diagnosis to death of any cause, sensored at last last follow-up date; recurrence-free survival was defined as time from date of diagnosis to any failure, sensored at the last follow-up date. Death without failure is considered as competing risk. The Kaplan-Meier method was used to estimate the probabilities for OS, RFS, DSS, local control (LC), regional control (RC) and distant control (DC) for the entire cohort and then, in an exploratory manner, for the individual head and neck subsites. Analyses were performed for individual subsites to minimize potential difference in clinical behavior atributable to variation in anatomy and tumor biology. The analyzed subsites included oral cavity (OC), oropharynx, and larynx. Hypopharynx was excluded from specific subsite analysis due to very small numbers in our cohort. Survival and control rates between metformin users with non-metformin users were compared by log-rank test. A two-sided test was applied with alpha set at 0.05 for statistical significance. Cox proportional hazards regression models were used for univariable (UVA) and and multivariable analysis (MVA). Variables included for MVA were clinical stage and comorbidity due to their clinical importance. Hazard ratios and corresponding 95% confidence intervals were provided. The proportionality assumption was tested using residuals. All statistical analyses were conducted using SAS 9.3 and R.

## Results

### Study population

There were 329 patients with T2DM eligible for inclusion, of which 195 (59%) were taking metformin at the time of presentation. The median age of the cohort was 67.9 years. Larynx was the most common cancer subsite (36%), followed by oral cavity (36%), oropharynx (24%), and hypopharynx (4%). Table 1 summarizes the baseline characteristics of the patient cohort. Statistically significant differences between the T2DMM+ group and T2DMM- groups included alcohol consumption history, with heavier drinkers in the non-metformin cohort (*p* = 0.029); greater pathologic extracapsular spread (ECS) was observed in the metformin cohort (*p* = 0.04) and higher Charlson Comorbidity Index (CCI) score in the non-metformin cohort (*p* = < 0.001). The mean and median follow up times for the entire cohort were 3.1 and 2.7 years, respectively.

**Table 1:** Baseline characteristics of study population according to metformin use

Co-variate	Full Sample (*n* = 329)	Non-Metformin (*n* = 134)	Metformin (*n* = 195)	*p*-value
Primary Site (%)				0.71
Oral cavity	117 (36)	49 (37)	68 (35)	
Oropharynx	80 (24)	36 (27)	44 (23)	
Hypopharynx	13 (4)	5 (4)	8 (4)	
Larnyx	119 (36)	44 (33)	75 (38)	
Age				0.9
Mean (sd)	67.4 (9.7)	67.6 (9.7)	67.3 (9.8)	
Median (Min,Max)	67.9 (40.9,89.9)	68.3 (42.6, 89.9)	67.6 (40.9, 89.6)	
Treatment Modality (%)				0.67
Surgery	73 (22)	30 (22)	43 (22)	
Adjuvant Rad	38 (12)	14 (10)	24 (12)	
Adjuvant Chemo/Rad	8 (2)	1 (1)	6 (3)	
Primary Rad	159 (48)	66 (49)	93 (48)	
Primary Chemo/Rad	52 (16)	23 (17)	29 (15)	
Extracapsular spread (%)				0.04*
Absent	52 (84)	24 (96)	28 (76)	
Present	10 (16)	1 (4)	9 (24)	
Missing	267	109	158	
Perineural invasion (%)				0.49
No	47 (59)	22 (65)	25 (56)	
Yes	32 (41)	12 (35)	20 (44)	
Missing	250	100	150	
CCI (%)				< 0.001*
0	9 (3)	9 (7)	0 (0)	
1	186 (57)	61 (46)	125 (64)	
2	78 (24)	36 (27)	42 (22)	
3+	56 (17)	28 (21)	28 (14)	
Smoking (%)				0.69
Never	74 (23)	32 (24)	42 (22)	
Current/Ex	252 (77)	101 (76)	151 (78)	
Missing	3	1	2	
Drinking (%)				0.029*
Never/Light	188 (63)	67 (55)	121 (68)	
Moderate/Heavy/Ex	111 (37)	54 (45)	57 (32)	
Missing	30	13	17	
Clinical Stage (%)				0.91
Early 0/I/II	133 (41)	54 (41)	79 (42)	
Late III/IV	190 (59)	79 (59)	111 (58)	
Missing	6	1	5	
Stage cN (%)				0.42
N0/Nx	190 (59)	77 (58)	113 (59)	
N1	23 (7)	7 (5)	16 (8)	
N2	100 (31)	46 (35)	54 (28)	
N3	11 (3)	3 (2)	8 (4)	
Missing	5	1	4	
Stage cT (%)				0.63
T0/Tx	1 (0)	0 (0)	1 (1)	
T1	81 (25)	28 (22)	53 (28)	
T2	101 (32)	45 (35)	56 (29)	
T3	77 (24)	33 (25)	44 (23)	
T4a/4b	60 (19)	24 (18)	36 (19)	
Missing	9	4	5	
Staging cM (%)				0.52
M0	327 (99)	134 (100)	193 (99)	
M1	2 (1)	0 (0)	2 (1)	
Follow-up time				0.69
Mean (sd)	3.1 (2.1)	3 (2.2)	3.1 (2.1)	
Median (Min, Max)	2.7 (0,8.8)	2.7 (0,8.8)	2.8 (0,8.4)	

* indicates statistical significance

### All HNSCC patients with T2DM

**Fig. 1:**
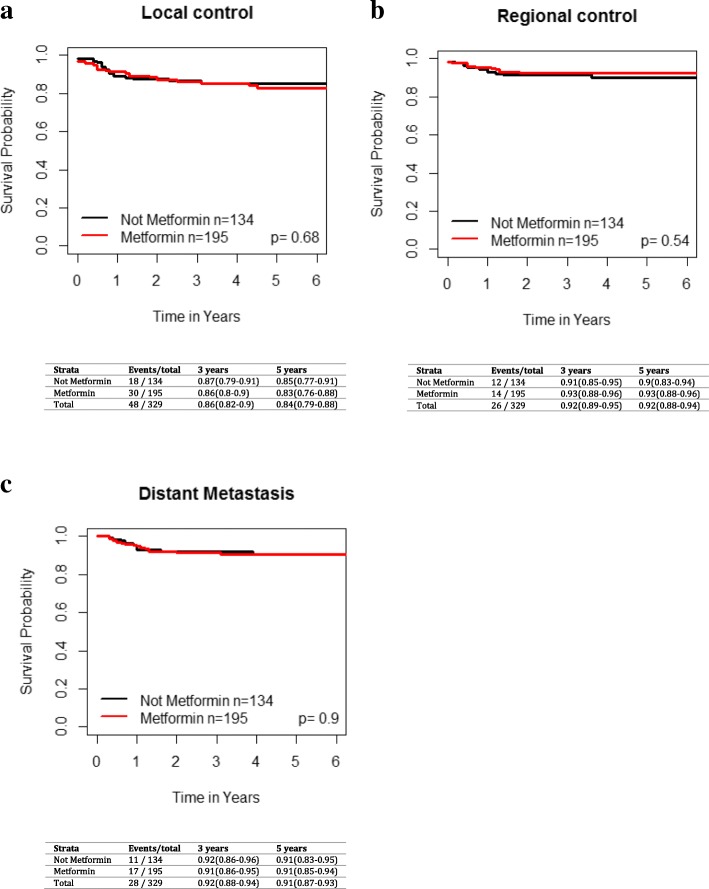
Impact of metformin use in T2DM across all subsites on **a**) local failure, **b**) regional failure, and **c**) distant failure. Kaplan-Meier analysis with associated p values for the entire grouping is demonstrated

**Fig. 2:**
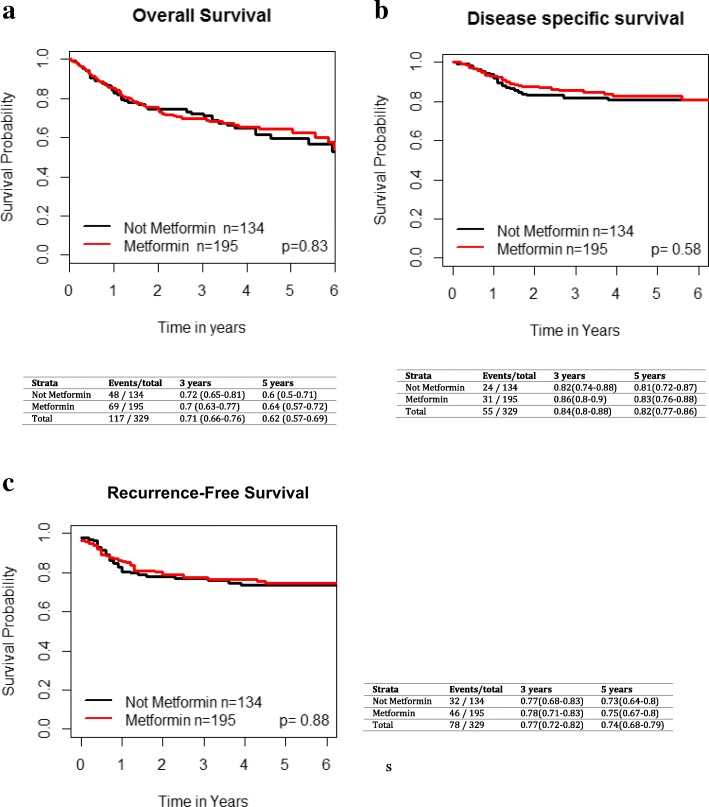
Impact of metformin use in T2DM patients across all subsites on **a**) overall survival, **b**) disease specific survival and **c**) recurrence-free survival. Kaplan-Meier analysis with associated *p* values is demonstrated

On UVA, metformin use was not significantly associated with improved LC (HR 1.13 [95% CI 0.63–2.02], *p* = 0.68), regional control (HR 0.78 [95% CI 0.36–1.69], *p* = 0.54) or distant control (HR 1.05 [95% CI 0.49, 2.24], *p* = 0.9) (Fig. 1). For the entire cohort, the 3 and 5 year OS was 71% [95% CI 66–76%] and 62% [95% CI 57–69%], respectively. The 3 and 5 year OS, RFS and DSS for the overall cohort based on metformin use are shown in Fig. 2. There was no significant difference in overall survival (HR 1.04 [95% CI 0.72–1.5], *p* = 0.83), recurrence-free survival (HR 1.04 [95% CI 0.66–1.62], *p* = 0.88) and disease-specific survival (HR 1.16 [95% CI 0.68–1.98], *p* = 0.58) between metformin and non-metformin users.

### Exploratory analysis of T2DM patients with Oral cavity cancers

**Fig. 3:**
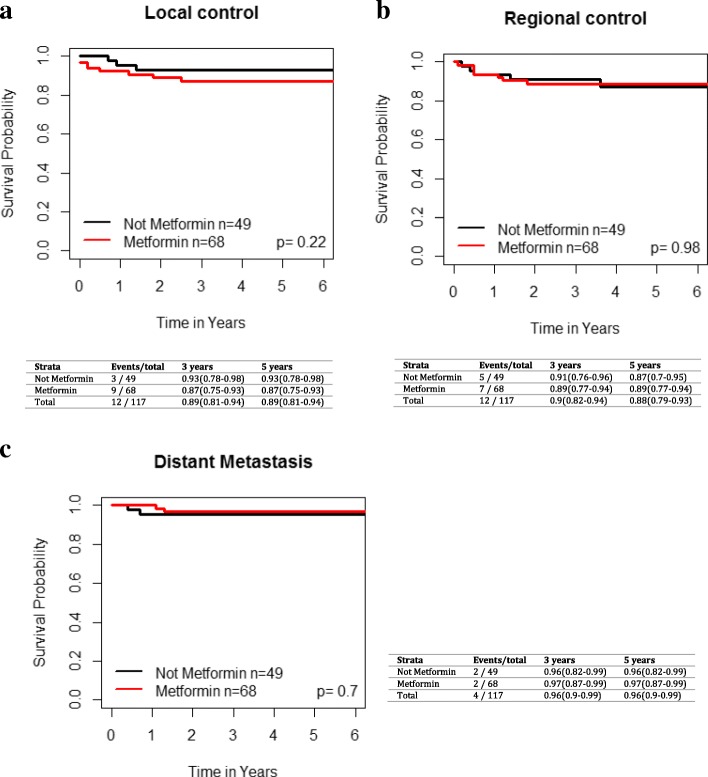
Impact of metformin use in T2DM patients with oral cavity cancer on **a**) local failure, **b**) regional failure, and **c**) distant failure. Kaplan-Meier analysis with associated *p* values is demonstrated

**Fig. 4:**
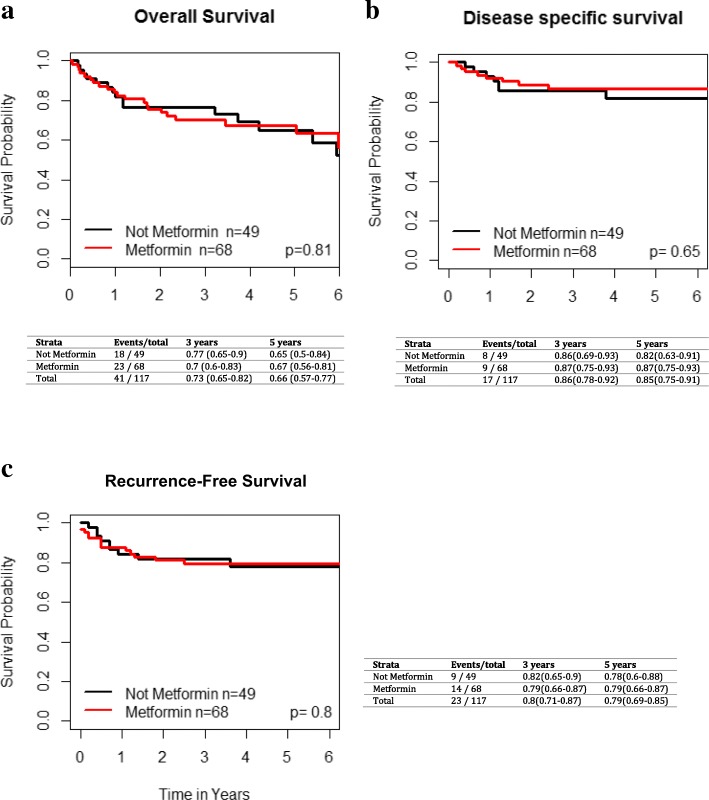
Impact of metformin use in T2DM patients with oral cavity cancer on **a**) overall survival, **b**) disease specific survival and **c**) recurrence-free survival. Kaplan-Meier analysis with associated p values is demonstrated

In the OC subgroup, there were 49 (42%) non-metformin users and 68 (58%) metformin users. On UVA, metformin use was not significantly associated with improved LC (HR 0.46 [95% CI 0.12–1.76], *p* = 0.24), regional control (HR 1.02 [95% CI 0.33–3.15], *p* = 0.98) or distant control (*p* = 0.69, HR 1.47 (95%CI 0.22–9.85) during the follow up period (Fig. 3). There were no significant differences in OS (*p* = 0.8, HR 1.08 [95% CI 0.58–2.01]), RFS (*p* = 0.8, HR 0.9 [95% CI 0.39–2.05]) or DSS (*p* = 0.63, HR 1.26 [95% CI 0.49–3.22]) between T2DMM+ and T2DMM- groups (Fig. 4).

### Exploratory analysis of T2DM patients with oropharynx

**Fig. 5:**
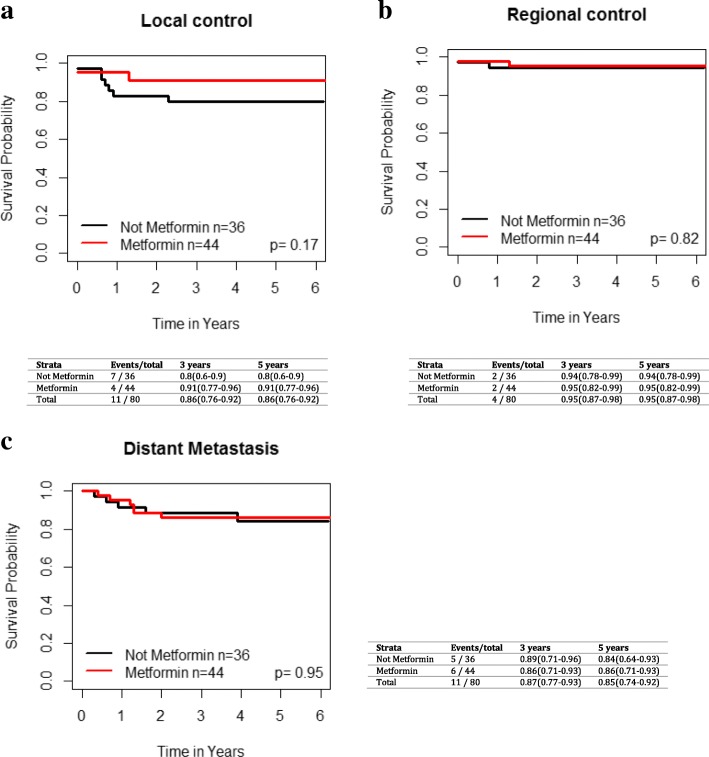
Impact of metformin use in T2DM patients with oropharynx cancer on **a**) local failure, **b**) regional failure, and **c**) distant failure. Kaplan-Meier analysis with associated p values is demonstrated

**Fig. 6:**
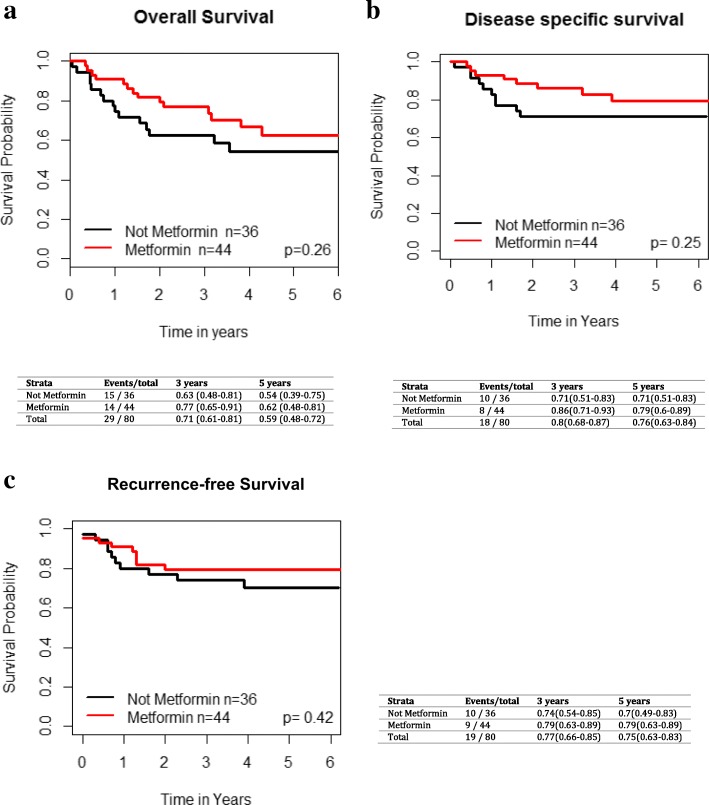
Impact of metformin use in T2DM patients with oropharynx cancer on **a**) overall survival, **b**) disease specific survival and **c**) recurrence-free survival. Kaplan-Meier analysis with associated p values is demonstrated

There were 44 oropharynx cancer (OPC) patients on metformin and 36 diabetic OPC patients not taking metformin. Metformin use in T2DM OPC patients was not significantly associated with improved LC (HR 2.31 [95% CI 0.68–7.79], *p* = 0.18), regional control (HR 1.25 [95% CI 0.18–8.68], *p* = 0.82) or distant control (HR 1.04 [95% CI 0.32–3.34], *p* = 0.95) (Fig. 5). The 3 and 5 year OS, RFS, and DSS for OP patients are shown in Fig. 6. There were no significant differences in OS (*p* = 0.26, HR 1.52 [95% CI 0.73–3.16]), RFS (*p* = 0.42, HR 1.44 [95% CI 0.59–3.51]), and DSS (*p* = 0.25 HR 1.72 [95% CI 0.69–4.32]) between the two cohorts of OPC patients on UVA. Given the differences in biology of HPV-related and HPV-unrelated OPC, we performed an additional exploratory analysis of outcomes in metformin and non-metformin group based on p16 status, acknowledging that the results need to be interpreted with caution given the limited numbers. There were a total of 67 OPC patients with known HPV status, including 47 p16+ patients (70%) and 20 p16 negative patients (30%). Within the p16+ cohort of T2DM OPC patients, there were no differences between the metformin and non-metformin patients in terms of OS (*p* = 0.59), RFS (*p* = 0.71), DSS (*p* = 0.62), local control (*p* = 0.74), regional control (*p* = 0.43) and distant control (*p* = 0.76). For p16 negative OPC diabetic patients, similarly, there were no differences between metformin and non-metformin groups in terms of OS (*p* = 0.14), RFS (*p* = 0.56), DSS (*p* = 0.32), local control (p = 0.56), regional control (*p* = 0.5), and distant control (*p* = 0.29). Of note, there were very few local and regional failures in this population.

### Exploratory analysis of T2DM patients with larynx Cancer

**Fig. 7:**
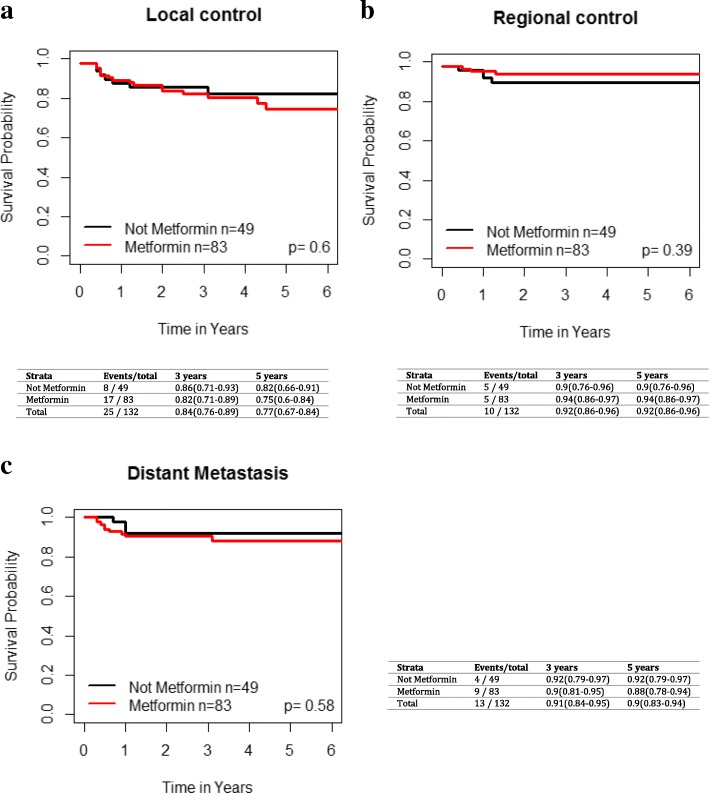
Impact of metformin use in T2DM patients with larynx cancer on **a**) local failure, **b**) regional failure, and **c**) distant failure. Kaplan-Meier analysis with associated p values is demonstrated

**Fig. 8:**
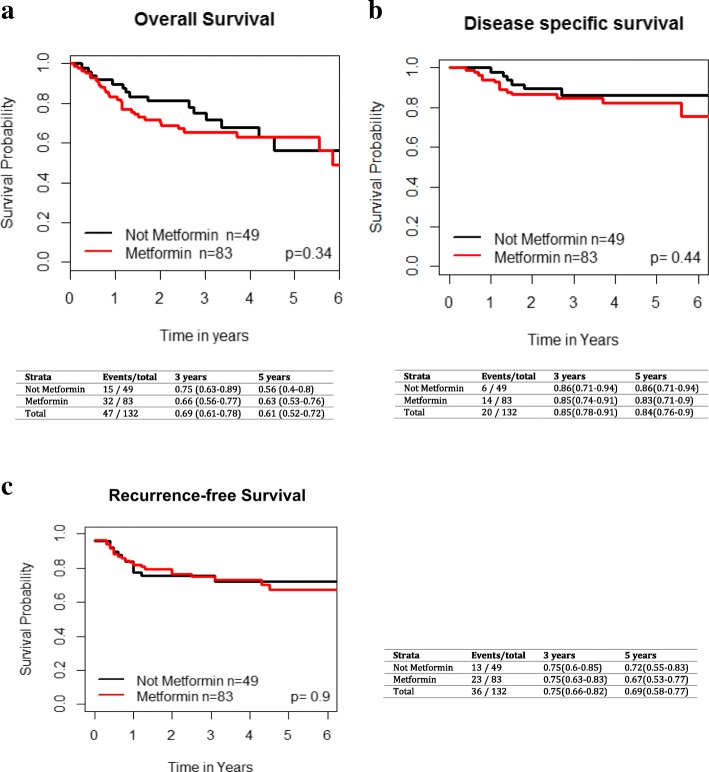
Impact of metformin use in T2DM patients with larynx/hypopharynx cancer on **a**) overall survival, **b**) disease specific survival and **c**) recurrence-free survival. Kaplan-Meier analysis with associated p values is demonstrad

There were 75 larynx cancer patients with T2DM on metformin and 44 T2DM patients not on metformin. The 3 and 5 year local, regional, and distant control rates for the T2DM laryngeal cancer patients based on metformin use are presented in Fig. 7. Metformin use was not significantly associated with improved LC (*p* = 0.87, HR 0.93 [95% CI 0.4–2.19]), regional control (*p* = 0.24, HR 2.17 (95% CI 0.59–8)) or distant control (*p* = 0.78, HR 0.83 [95% CI 0.21–3.22]). There were no significant differences in OS (*p* = 0.28, HR 0.69 [95% CI 0.35–1.36]), RFS (*p* = 0.75, HR 1.12 [95% CI 0.55–2.3]), and DSS (*p* = 0.73 HR 0.83 [95% CI 0.29–2.38]) between the two cohorts of laryngeal cancer patients upon univariate analysis (Fig. 8).

## Discussion

Metformin was originally noted to have potential oncologic benefits in an observational study published in 2005, which reported that its use was associated with a decreased incidence of cancer [20]. One of the proposed mechanisms for its anti-neoplastic effects is the reduction of systemic levels of insulin and insulin-like growth factor-1 (IGF-1), thus lowering the mitogenic activity of hyperinsulinemia [21–23]. In addition, it is hypothesized that metformin inhibits the mTOR pathway and activation of AMP-activated protein kinase (AMPK), leading to the blockade of cell proliferation in cancer cells [23–25]. Dysregulation of mTOR signaling has been identified as a targetable oncogenic pathway in HNSCC [24]. In certain breast cancers, the cell adhesion protein CD24+ plays a role in the development of distant metastases and is suppressed by metformin in vitro [26]. CD24+ has also been associated with aggressive features in HNSCC cell lines [27]. Studies included in a recent systematic review of the anti-tumor effects on head and neck cancer cell lines reported that metformin plays a role in proliferation inhibition, apoptosis, G0/G1 cell cycle arrest, and carcinogenic pathway protein regulation [28].

Given that metformin is a low cost, well-tolerated medication that has minimal toxicity, it would be an ideal anti-cancer agent. While some clinical studies have demonstrated that the use of metformin results in potentially increased overall survival and decreased incidence of cancer in multiple sites and cell types, such as colon, breast and liver [9, 10, 13, 29–31], there are contradicting randomized controlled trials and observational studies that have failed to demonstrate an unequivocally beneficial effect of metformin use on cancer survival [14, 32–34]. In HNSCC, the data are limited and results of existing studies are similarly mixed, particularly with respect to reporting the effects of metformin on outcomes such as survival or recurrence. One of the earliest observational reports in the head and neck cancer literature was by Skinner et al. who found that patients with oral cavity, oropharynx (HPV status not reported), larynx and hypopharynx cancer on metformin at the time of radiation had a lower locoregional recurrence rate compared to controls matched for tumor and nodal stage, surgical margin status, and *TP53* status (*p* = 0.04) [7]. However, the results need to be interpreted with caution with only 10 patients in the metformin group. Sandulache et al. examined the observational association between metformin use and outcomes in glottic and supraglottic laryngeal carcinoma [15]. They included 21 diabetic patients on metformin and 22 diabetic patients not taking metformin. In these relatively small groups, metformin use was marginally significant but of a great magnitude of benefit in improving overall survival (OS) (OR, 3.0; 95% CI, 1.0–8.4; *p* = .04); of note, there was no difference in RFS between the two groups. Given the limited number of patients and outcomes (i.e. death) the multivariable analysis must be interpreted with caution. It could not be excluded that the deaths were actually due to non-cancer reasons (e.g. cardiovascular disease) that could be linked to metformin use (e.g., use of metformin may represent better T2DM care). Patients seeking care for their diabetes and taking metformin may seek out other healthy behaviors to modify their risk, potentially resulting in skewed survival results [35]. In addition, comorbidities were not assessed. While HbA1c levels were similar between groups, it is difficult to ascertain whether the improved survival is purely attributable to the effect of metformin without accounting for the co-morbidities. The primary endpoints of their study were OS and RFS; information specifically regarding distant and locoregional control were not reported. Similar to our results, Yen et al. using a sample of diabetic patients enrolled in the National Health Insurance Program of Taiwan reported that while metformin use was associated with a reduction in the incidence of developing head and neck cancers, there was no significant difference in overall survival between patients with diabetes in the metformin positive (*n* = 195) and metformin negative (*n* = 290) cohorts who subsequently developed head and neck cancer (sites included oral cavity, oropharynx, larynx, hypopharynx, salivary gland, nasopharynx and nasal/sinus)^16^. In the most recent study, Spratt et al. performed a retrospective review of 184 diabetic OPC patients treated with primary radiation, of which 102 were on metformin [36]. They reported improved distant metastasis-free survival for the cohort of patients on metformin; however, there was no difference observed for 5-year local and regional failure-free survival. There was no difference between metformin and non-metformin in terms of proportion of patients that were HPV positive. Our study failed to demonstrate a benefit of metformin use in OPC patients, for either the HPV positive and negative groups. Similarly, across oral cavity and larynx cancer patients there was no benefit of improved survival with metformin use amongst diabetic patients.

In terms of comparing clinical results with preclinical studies, the latter utilized much higher doses of metformin than the conventional dose used in the treatment of diabetes [8, 37, 38]. Considering the pharmacokinetics, the actual quantity of metformin delivered to cancer cells may be insufficient to truly demonstrate its anti-proliferative effects [39, 40]. These shortcomings of the studies render the results less generalizable to human subjects who are taking metformin at a much lower dose. There are some limitations of the current study that needs to be considered. Patients included in this study were defined as metformin-users based on use at the time of diagnosis, but the duration of use, dosage, medication compliance, and medication changes were not incorporated into analysis, as this information was not available from retrospective chart review. As reported by Suissa et al., this may lead to time-related biases [33]. For example, Suissa et al. describe immortal time bias which occurs when the time prior to the medication exposure is misclassified and may skew results towards risk reduction [33]. Time-related bias issues are not addressed by many authors reporting differential outcomes between metformin users and diabetic non-metformin users. Confounding factors including renal and hepatic function affecting drug metabolism and glucose control were also not included. Furthermore, the prescription of metformin over injectable insulin or other oral hypoglycemic drugs may indicate diabetic severity, which is known to affect survival outcomes. Lastly, while the study is one of the largest institutional series, it is limited by the number of diabetic patients as well as the number of patients lost to follow up. Given the small sample size in the subset analyses the results have to be interpreted with caution and while metformin was not associated with recurrence or survival in our cohort this may be due lack of power to demonstrate such a finding. However, other studies mentioned in the discussion that reported an effect of metformin on outcomes also had relatively small sample size. In addition, the metforming usage was obtained via retrospective review, and we are unable to explore the potential influence of the duration, dosage and the intensity of metformin on outcome of head and neck cancer patients.

The impact of a new agent with a favorable side effect profile for effective adjuvant therapy in HNSCC would be enormous. However, the clinical data thus far has failed to definitively demonstrate a significant effect on outcomes in metformin users. This study reinforces prior work that suggest this drug may not be useful in HNSCC patients overall or by subsite.

## Conclusion

In our HNSCC cohort, the use of metformin was not associated with improved overall survival, disease-specific survival, local, regional, or distant control overall or within any subsite. Additional prospective multi-centre studies are needed to further elucidate the role of metformin in this disease.

## Data Availability

Dr. David P. Goldstein and Dr. Daniel J. Lee had full access to the study and take full responsibility for the integrity and accuracy of data analysis.
